# Beyond surgical radicality in intramedullary spinal cord metastases: neurological function and systemic disease burden drive patient outcomes

**DOI:** 10.1007/s11060-025-05119-5

**Published:** 2025-06-30

**Authors:** Meltem Ivren, Dilber Yalman, Basem Ishak, Sebastian Ille, Sandro M. Krieg, Pavlina Lenga

**Affiliations:** 1 Department of Neurosurgery, Heidelberg University, Im Neuenheimer Feld 400, 69120 Heidelberg, Germany; 2 Department of Neurosurgery, ATOS Klinik Wiesbaden, Wiesbaden, Germany

**Keywords:** ISCM, Intramedullary metastases, Neurosurgical oncology, Extent of resection, Spinal metastases

## Abstract

**Purpose:**

Intramedullary spinal cord metastases (ISCM) are rare, clinically challenging lesions with limited evidence-based guidance. Optimal surgical management remains controversial, particularly regarding the ideal extent of resection (EOR) and associated prognostic factors. This study systematically evaluates perioperative outcomes, neurological function, and short-term survival according to biopsy-only, subtotal, or gross total resection (GTR) approaches.

**Methods:**

This retrospective single-center study included 16 patients treated surgically for histologically confirmed ISCM between 2015 and 2024. Patients were stratified by surgical extent (biopsy, subtotal, or total resection). Outcomes included perioperative complications, neurological function, and 90-day survival. A literature review of surgical ISCM series (≥ 5 cases) was also performed.

**Results:**

Sixteen patients with a median age of 59 years (56% male) were included. Thoracic lesions predominated (56%). Surgical complications were seen in 19% of the cases, however no irreversible neurological injury and no intraoperative mortality or transfusion occured. Early mortality was lowest following GTR (13%) compared to subtotal (60%) or biopsy-only (33%) approaches (*p* = 0.015). However, multivariable regression showed that EOR alone was not independently predictive of short-term survival (*p* = 0.834), indicating patient selection bias. Neurological function remained stable or improved in all of cases, irrespective of resection extent.

**Conclusion:**

Surgical management of ISCM can be performed safely with minimal neurological morbidity, achieving symptom stabilization or improvement. Although GTR was associated with favorable short-term survival, systemic disease burden and postoperative neurological function are stronger prognostic factors. Therefore, surgical decisions should prioritize functional preservation and patient selection based on disease extent and overall health.

## Introduction

Intramedullary spinal cord metastases (ISCM) are rare yet clinically critical, representing approximately 1–3% of intramedullary spinal tumors and affecting around 0.4% of patients with systemic malignancies [1, 2]. Their rarity and associated neurological impairment pose major diagnostic and therapeutic challenges [3]. Clinically, ISCM typically present with rapidly progressing motor, sensory, or autonomic deficits, impacting quality of life [4]. Historically, management relied on systemic treatments due to concerns regarding surgical feasibility, comorbidities, and generally poor prognosis [3, 5].

Recent advancements in microsurgical techniques, intraoperative monitoring, and neuroimaging have improved feasibility and safety of surgery [5, 6]. This has shifted care toward surgical management and sparked debate over optimal patient selection and surgical strategy—particularly the trade-off between maximal tumor resection and neurological preservation [3, 7–9]. Some studies support gross total resection (GTR) for local disease control [10], while others report no clear survival advantage from aggressive resections [11–13]. Additionally, predictive markers for surgical outcomes remain poorly defined, complicating clinical decision-making [3, 14].

Given these uncertainties, comparative analysis of ISCM surgical strategies is urgently needed. This study evaluates perioperative outcomes, complications, functional improvements, and short-term survival associated with biopsy-only, partial resection, and GTR. We also aim to identify prognostic factors affecting survival. Our goal is to provide evidence-based guidance for multidisciplinary management of ISCM.

## Methods

### Study design

This retrospective study analyzes data collected from our institution’s database between January 2015 and October 2024. We examined demographics, comorbidities, clinical progression, and mortality to assess treatment outcomes among patients undergoing biopsy, partial or total resection of an ISCM. Ethical approval was granted by our local ethics committee (approval number 880/2021). The study adhered to the Declaration of Helsinki.

### Inclusion and exclusion criteria

Patients with histologically confirmed ISCM who underwent surgery during the study period were included. Preoperative MRI was required in all cases to evaluate tumor extent and potential neurological involvement. Postoperative MRI was required to determine the EOR. Exclusion criteria comprised primary spinal tumors, non-metastatic lesions, incomplete records, exclusively non-surgical treatment or inadequate imaging.

### Demographic, clinical and surgical data

We analyzed age, sex, BMI, ASA classification, the age-adjusted Charlson Comorbidity Index (CCI) [15, 16], primary tumor peri- and postoperative complications, hospital/ ICU length of stay, in hospital and 90-day mortality.

Per CCI, metastatic tumors add 6 points. However, to better reflect overall comorbidity, we excluded ISCM itself from scoring and added 6 points only for metastases beyond the spinal cord.

Surgical and anesthesiology records provided data on EOR (biopsy, subtotal, total), surgery duration, blood loss, and transfusions.

Clinical presentation and course were evaluated using the Motor Score (MS) and Modified McCormick Scale (mMCS). The MS, based on ASIA guidelines, sums muscle strength scores (0–5) from five key myotomes per limb, totaling 0–100 [17]. The mMCS assesses neurological and ambulatory function on a 5-point scale, from I (normal or mild symptoms) to V (severe disability or complete dependence) [18].

### Indications for surgery

All cases were reviewed in a multidisciplinary tumor board. Surgical decisions were individualized, aiming at neurological preservation, symptom relief, and quality of life improvement. Surgery was offered for acute/progressive deficits from spinal cord compression, refractory pain, or unclear diagnosis needing tissue confirmation.

GTR was preferred for solitary spinal lesions, good baseline function (KPS ≥ 70%), limited systemic disease, and resectable imaging features.

Biopsy or subtotal resection was pursued in patients with multifocal metastases, widespread disease, or poor neurological status, minimizing morbidity. Non-operative care was reserved for those with advanced systemic illness or poor clinical condition.

### Statistical analysis

Statistical analysis was conducted using SPSS (version 27, IBM Corp.). Normality was assessed via Shapiro-Wilk. Categorical data are presented as counts/ percentages; continuous data as mean ± SD. Chi-squared or Fisher’s exact test was used for categorical comparisons. Spearman’s correlation assessed associations with tumor volume. Binary logistic regression identified predictors of 90-day mortality. Kaplan-Meier analyses assessed survival factors for survival. Significance was set at *p* ≤ 0.05.

### Literature review

We reviewed the last 30 years of ISCM surgical literature using PubMed. Keywords included “intramedullary metastasis,” “intramedullary spinal cord metastasis,” and “ISCM.” Included studies were English, peer-reviewed, on humans, and reported ≥ 5 ISCM surgical cases. We excluded animal studies, non-intramedullary metastases, non-English texts, unavailable full-texts, and reviews lacking patient data.

## Results

### Patient cohort and surgical groups

Sixteen patients with ISCM were included (mean age 51 years, 56% male). The primary tumor types were varied (lung 19%, melanoma 19%, lymphoma 13%, breast 6%, others 6% each, unknown 19%). Lesions were located in the cervical cord in 19%, thoracic in 56%, and conus medullaris in 25%. Patients were stratified by surgical intervention: 3 underwent biopsy only, 5 had subtotal (partial) resection, and 8 had GTR. Notably, all three biopsy-only patients had disseminated disease (e.g. leptomeningeal spread or multiple spinal metastases), prompting a limited surgical approach. In the partial resection group, acute spinal cord injury symptoms were the reason for surgery in all cases. In the total resection group, acute symptoms led to surgery in 5 out of 8 cases. In two cases, the indication for surgery was to obtain tissue for pathological analysis. In one particular case, an additional epidural metastasis was scheduled for resection, and - at the patient’s explicit request - the intramedullary metastasis was also removed, despite the patient being fully informed of and accepting the associated risks. Preoperatively, most patients were in moderate neurological condition: the mean preoperative Karnofsky Performance Status (KPS) was approximately 85–90%, and the modified McCormick grade was I–II in 38% of patients, III in 12%, and IV–V in 50%. There were no significant differences in baseline age or comorbidities between the surgical groups (comp. Table 1).

**Table 1:** Baseline characteristics

	Overall co-hort, n = 16	Biopsy only	Partial resection	Total resection	*p* value
		n = 3	n = 5	n = 8	
**Age,y (mean, SD)**	51 (17)	42 (23)	60 (4)	49 (19)	0.124
**Sex (n, %)**					0.672
Male	9 (56)	1 (33)	3 (60)	5 (63)	
Female	7 (44)	2 (67)	2 (40)	3 (38)	
Charlson Comorbidity Index (mean, SD)	4 (3)	4 (5)	5 (3)	2 (1)	0.256
**Karnofsky** Performance status	*66 (21)*	63 (31)	54 (17)	75 (17)	0.194
(mean, SD)					
**ASA class (n,%)**					0.319
I	*0 (0)*	0 (0)	0 (0)	0 (0)	
II	*2 (13)*	0 (0)	0 (0)	2 (25)	
III	*14 (87)*	3 (100)	3 (0)	6 (75)	
IV	*0 (0)*	0 (0)	0 (0)	0 (0)	
V	*0 (0)*	0 (0)	0 (0)	0 (0)	
**Localization (n,%)**					0.911
Cervical	3 (19)	1 (33)	1 (20)	1 (12)	
Thoracal	9 (56)	1 (33)	3 (60)	5 (63)	
Conus medullaris	4 (25)	1 (33)	1 (20)	2 (25)	
**Primary tumor (n, %)**					0.294
Lung	3 (19)		2 (40)	1 (13)	
Melanoma	3 (19)		1 (20)	2 (25.0)	
Lymphoma	2 (13)		1 (20)	1 (13)	
- thereof plasmocytoma	1 (6)		1 (20)		
ALL	1 (6)			1 (13)	
Breast	1 (6)			1 (13)	
Ovary	1 (6)	1 (33)			
Sarcoma	1 (6)		1 (20)		
Urothelial	1 (6)			1 (13)	
Unknown	3 (19)	2 (67)		1 (13)	
**Preoperative MS score (mean, SD)**	87 (18)	85 (22)	80 (23)	92 (14)	0.604
**Neurological symptoms**					0.172
Motor	11 (69)	2 (67)	4 (80)	5 (63)	
Sensory	11 (69)	1 (33)	3 (60)	7 (88)	
Sphincter/bladder dysfunction	7 (44)	1 (33)	2 (40)	4 (50)	
Gait ataxia	12 (75)	3 (100)	4 (80)	5 (63)	
Pain	12 (75)	2 (67)	3 (60)	7 (88)	
**Modified McCormick Score**					0.372
I	1 (6)	0 (0)	0 (0)	1 (12)	
II	5 (31)	1 (33)	1 (20)	3 (38)	
III	2 (13)	1 (33)	1 (20)	0 (0)	
IV	5 (31)	0 (0)	1 (20)	4 (50)	
V	3 (19)	1 (33)	2 (40)	0 (0)	
**Tumor volume [cm** ^**3**^ **]**	*2.9 (2.7)*	3.6 (3.8)	2.7 (2.0)	2.8 (3.2)	0.725
**mean (SD), n = 14**					
**Tumor volume**					0.769
**< 3cm** ^**3**^	*10 (71)*	1 (50)	3 (75)	6 (75)	
**≥ 3cm** ^**3**^	*4 (29)*	1 (50)	1 (25)	2 (25)	

The baseline parameters are given for each EOR group and the overall cohort. ASA class = American Society of Anesthesiologists classification, BMI = Body Mass Index, MS = Motor score of the American Spinal Cord Injury classification, SD = standard deviation, y = years, *P*-values indicate statistical significance between the groups

### Intraoperative parameters and hospital course

Operative duration varied by surgical approach (partial resection: ~146 min, biopsy: ~180 min, total resection: 206 min; *p* = 0.684). Mean intraoperative blood loss was lowest for biopsy-only (100 mL) and highest for total resection (443 mL), though differences were not statistically significant (*p* = 0.266), and no patient required transfusion. Average hospital stay was 9 ± 5 days (shortest after biopsy: 5 days, longest after partial resection: 12 days). ICU stays were brief or unnecessary. Surgical complications occurred in 3 patients (19%, one wound infection, one CSF leak), and mild medical complications in 1 patient (6%, urinary tract infections). No irreversible neurological injury occurred, confirming acceptable surgical safety. Table 2 provides a concise summary of patients’ surgical course.

**Table 2:** Surgical approaches and postoperative outcomes

	Overall cohort,	Biopsy only	Partial resection	Resection	*p* value
	n = 16	n = 3	n = 5	n = 8	
Duration of surgery	183 (77)	180 (52)	146 (86)	206 (79)	0.684
(min) (mean, SD)					
Intraoperative blood	300 (355)	100 (100)	200 (141)	443 (458)	0.266
loss (mean, SD)					
Intraoperative	0 (0)	0 (0)	0 (0)	0 (0)	
blood transfusion *(n, %)*					
Hospital stay (mean, SD)	9 (5)	5 (3)	12 (1)	10 (3)	0.079
ICU stay (mean, SD)	0 (1)	0 (0)	1 (1)	0 (0)	0.297
Mortality *(n, %)*					
- In hospital	1 (6)	0 (0)	1 (20)	0 (0)	0.309
− 90 days	4 (25)	1 (33)	3 (60)	1 (13)	**0.028**
Complications *(n, %)*	3 (19)	1 (33)	1 (20)	1 (13)	0.73
- Surgery-related	2 (13)	1 (33)	0 (0)	1 (13)	
- Other	1 (6)	0 (0)	1 (20)	0 (0.0)	
Complication what kind		Wound infection	Urinary tract infection	Postoperative CSF leak	

Intraoperative parameters as well as clinical course given for each EOR group and the overall cohort. Surgery-related complications encompass impaired wound healing. CSF leakage necessitating revision surgery and urinary tract infection. ICU = Intensive Care Unit, SD = standard deviation, *P*-values indicate statistical significance between the groups

### Perioperative mortality

One patient (6%), from the subtotal resection group, died during initial hospitalization (in-hospital mortality: subtotal resection 20%; biopsy and total resection 0%). By 90 days postoperatively, four patients had died (overall 90-day mortality 25%), with the poorest survival in the subtotal resection group (40% survival at 3 months), intermediate survival after biopsy (67%), and best survival after total resection (88%). However, this trend was not statistically significant (*p* = 0.834). Multivariable analysis found no significant predictors of 90-day mortality, including patient age, sex, comorbidity burden, tumor characteristics, or preoperative functional status (all *p* > 0.25). Shorter operative duration approached significance (*p* = 0.095), likely reflecting aggressive disease rather than surgical factors.

### Kaplan–Meier survival analyses stratified by preoperative and treatment factors

**Fig. 1:**
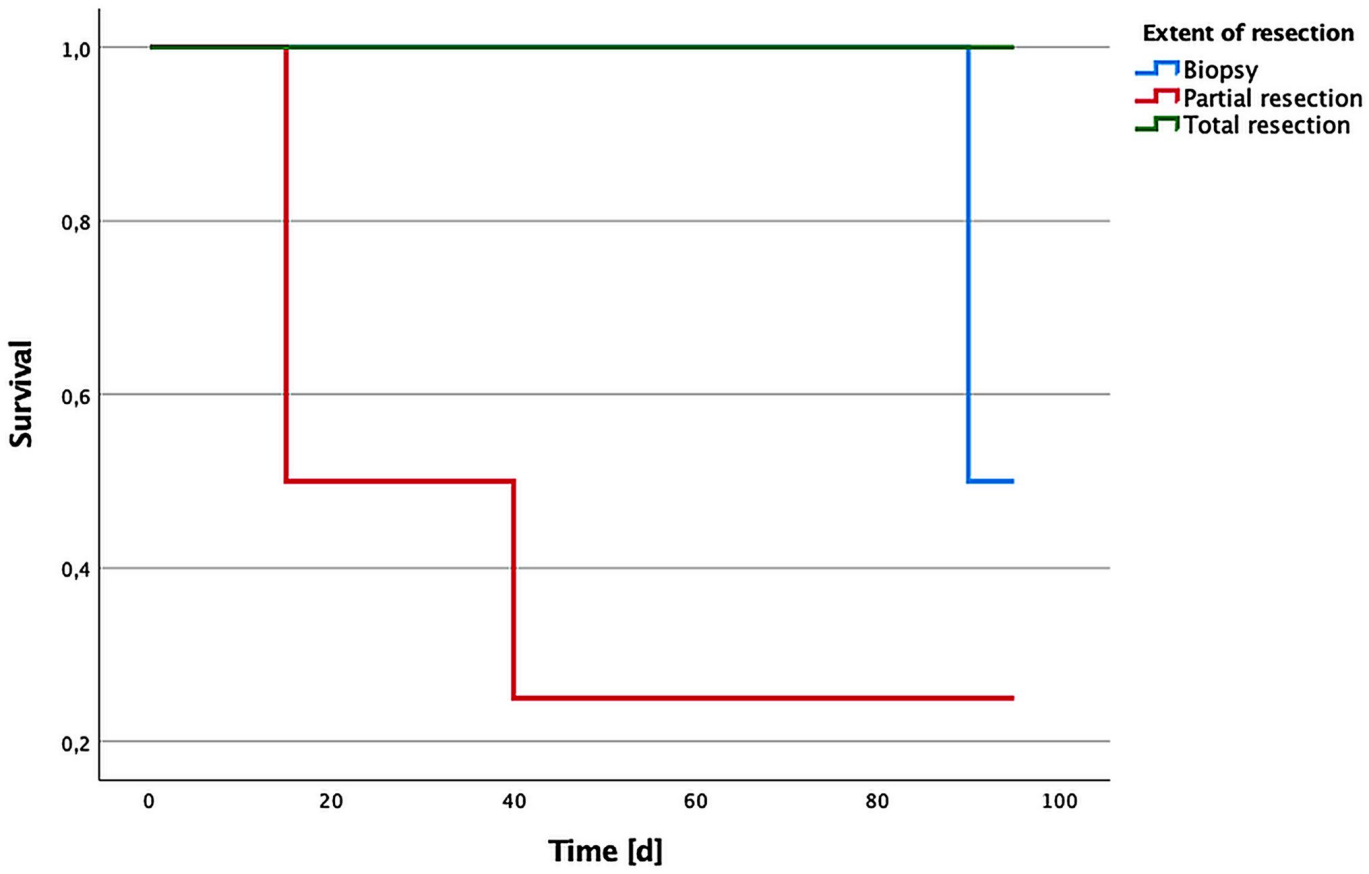
Kaplan–Meier Survival Curves Stratified by Extent of Resection in Patients with ISCM. Kaplan–Meier analysis illustrating postoperative survival in patients with intramedullary spinal cord metastases (ISCM), categorized by extent of surgical intervention. Patients who underwent total resection (green line) demonstrated the longest survival, while those with partial resection (red line) or biopsy only (blue line) showed significantly shorter survival durations. Time is displayed in days (d) post-surgery

Kaplan–Meier analysis showed no significant survival differences when patients were stratified by preoperative neurological status (KPS, motor score, McCormick grade), tumor volume, age, or occurrence of complications (all log-rank *p* > 0.5). Although patients with higher preoperative neurological function showed a trend toward better survival, this did not reach statistical significance, likely due to the small sample size. Survival curves for mild (McCormick grades I–III) and severe deficits (grades IV–V) largely overlapped, indicating no clear prognostic distinction. Tumor volume (categorized as small or large at a 3 cm cutoff) also did not significantly affect survival. Additionally, experiencing perioperative complications did not significantly worsen short-term survival outcomes. Although no statistically significant differences were observed among groups regarding neurological outcomes and complication rates, short-term survival was significantly better in patients who underwent GTR compared to those receiving subtotal resection or biopy (*p* = 0.015, Fig. 1).

### Neurological and functional outcomes

**Fig. 2:**
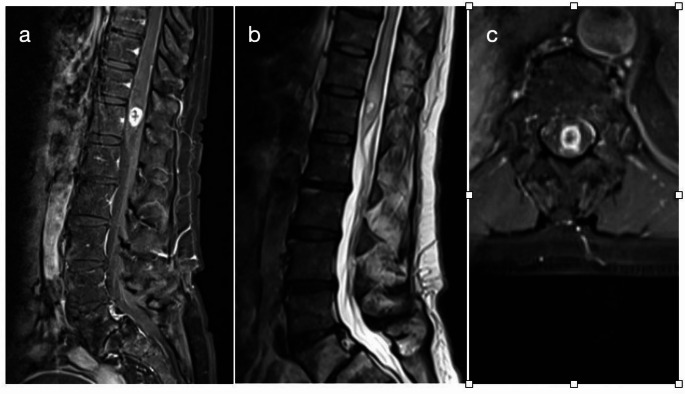
Preoperative MRI of a patient with conus medullaris ISCM. Sagittal T1-weighted image with gadolinium contrast (**A**), sagittal T2-weighted image (**B**), and axial T1-weighted image with gadolinium contrast (**C**) of a 61-year-old patient presenting with acute bladder dysfunction and emerging proximal leg weakness. A 12 × 6 mm intramedullary, contrast-enhancing lesion is visible at the level of the conus medullaris. The lesion was resected via T12 laminectomy and central myelotomy. The postoperative course was uneventful. Histopathological analysis confirmed a metastasis from non-small cell lung cancer (NSCLC)

Postoperative neurological status was largely maintained or improved compared to presentation. The mean KPS remained essentially unchanged from preoperative (mean ~ 87) to postoperative (~ 87) for the cohort. Similarly, quantitative motor scores showed minimal change (mean motor score Δ ≈ 0, with a mean increase of only + 0.13 ± 3 points), indicating no significant new motor deficits caused by surgery. In terms of ambulation and myelopathy grade, 50% of patients were graded McCormick IV–V pre- as well as postoperatively. 2 patients (13%) improved by one McCormick grade after surgery, while the majority (87%) remained stable. For instance, one patient improved from McCormick V to IV, and another improved from II to I (normal function). Despite these individual changes, there was no significant difference in the overall distribution of McCormick grades pre- vs. post-op (Spearman correlation with tumor volume was non-significant for both pre and postoperative scores, *p* = 0.374 and *p* = 0.335)​. Importantly, all patients who were ambulatory before surgery remained ambulatory after surgery, and no previously independent patient became permanently paraplegic due to the operation. In fact, the preservation of neurological function is highlighted by 100.0% of patients having either stable or improved McCormick grade at discharge. Figure 2 shows an MRI of the lumbar spine with a contrast-enhancing lesion in the conus medullaris measuring approximately 12 × 6 mm.

### Adjuvant therapies

Among the 16 patients in our cohort, adjuvant treatment data were available for 8 individuals. Eight patients were excluded from this analysis for the following reasons: no postoperative medical records were available in two cases; three patients died before adjuvant therapy could be initiated; one patient continued treatment at another institution; one refused further treatment; and in one case, therapy was withheld due to rapid clinical deterioration and explicit patient wish.

Among the 8 patients who received adjuvant therapy, radiotherapy was the most common modality and was administered in 7 cases. Five patients underwent spinal irradiation alone, with primary tumors including lung, breast, melanoma, urothelial carcinoma, and one case of cancer of unknown primary. Two patients received combined treatments: one patient with multiple myeloma was treated with radiotherapy alongside lenalidomide and dexamethasone, and another with acute lymphoblastic leukemia (ALL) received spinal irradiation and Blinatumomab. Immune checkpoint inhibition with Ipilimumab and Nivolumab was initiated in a patient with metastatic melanoma.

Adjuvant therapies were selected on an individual basis within a multidisciplinary setting, taking into account the primary malignancy, prognosis, neurological status, and patient preferences.

### Literature review

We reviewed 13 studies on surgical treatment outcomes in ISCM, each including ≥ 5 surgical cases. The number of cases ranged from 5 to 30 per study. Most were retrospective case series, with a few including systematic reviews. Primary tumor types varied; however, some studies focused on particular cancers, such as lung carcinoma (Wu et al.) [12]. Despite methodological variations and heterogeneous populations, most studies supported surgery for preserving or enhancing neurological function, though evidence on survival benefit was inconsistent. Median overall survival post-surgery ranged from 3 to 11.6 months. Several reports indicated that surgical management might improve survival over conservative treatments [12, 19, 20]. Nevertheless, GTR did not correlate with better outcomes than subtotal or partial resections [11, 12, 21]. Functional outcomes generally trended favorably: Iwasaki et al. and Gazzeri et al. reportedtmost patients maintained or improved neurological function postoperatively [11, 22]. Salvotti et al. similarly demonstrated neurological stability or improvement in 92% of patients [23]. A comprehensive summary of the reviewed literature is depicted in Table 3 [11, 12, 19–29].

**Table 3:** Summary of Peer-Reviewed studies reporting surgical resection in ≥ 5 patients with intramedullary spinal cord metastases (ISCM)

Study	Study type	Number of cases	Primary Tumor	Complications	Overall survival (mean/ median)
Schiff & O’Neill (1996) [24]	Retrospective analysis	5 surgical (out of overall 40 ISCM cases)	N/A	N/A	4 months with RT, 2 months without RT
Hejazi & Hassler (1998) [25]	Retrospective study	5 ISCM (out of 80 intramedullary tumors of other entities)	NS	- Neurological worsening in 2 cases	NS
Gasser et al. [21] (2005)	Retrospective case series	13	Various (7 adenocarcinomas, not further specified)	− 1 CSF leakage + bacterial meningitis - Neurological worsening in 2 patients	31 weeks
Sandalcioglu et al. (2005) [26]	Retrospective study	10 ISCM (out of 78 intramedullary tumors of other entities)	NS	2 CSF leakages in the overall cohort	NS for ISCM
Dam-Hieu et al. (2009) [20]	Retrospective study	13 surgical (out of 19 ISCM)	Various (mostly lung, *n* = 5 and breast, *n* = 2)	NS	6.1 months
Wilson et al. (2012) [27]	Retrospective study	9	Breast: *n* = 4 Lung: *n* = 3 melanoma: *n* = 2	No surgical complication	6.4 months
Goyal et al. (2015) [19]	Retrospective study	8	Various; Lung: *n* = 5	NS	4.5 months
Payer et al. (2015) [28]	Retrospective study	22	Various; Lung: *n* = 5, Brain: *n* = 3, Breast: *n* = 3	No complications	11.6 months
Gazzeri et al. (2021) [11]	Retrospective study	30	Various Lung: *n* = 9, Breast: *n* = 6	− 1 superficial wound infection − 1 CSF leak − 1 brain hemorrhage − 1 epidural hematoma - Neurogical worsening in 6 patients	9.9 months
Wu et al. (2022) [12]	Retrospective study and systematic review	6	Lung: *n* = 6	1 death from pulmonary embolism, other surgical complications NS	5 months
Iwasaki et al. (2023) [22]	Multicenter retrospective study	29	Various Colorectal: *n* = 9 Lung: *n* = 7 Renal cell: *n* = 5 Breast: *n* = 2	No surgical complications	72.4% survived after 6 months
Kritikos et al. (2024) [29]	Retrospective Case series	9	Various: Lung: *n* = 2, Renal: *n* = 2, Melanoma: *n* = 2	Neurological worsening in 1 patient, no other surgical complications	7 months
Salvotti et al. (2024) [23]	Single-center retrospective case series and review	8 ISCM (out of 13 intradural tumors including extramedullary cases)	Various Lung: *n* = 2 Breast: *n* = 2	Neurological worsening in 1 patient, no other surgical complications	5 months

Literature review: the number of cases refers to surgically treated ISCM cases. If the analyzed cases were a subset of a broader cohort (e.g. intramedullary tumors), this information was specified. Primary tumor origins include only the most frequent cases of each cohort. Complications include surgical complications such as wound infections as well as postoperative neurological worsening. Overall survival was given as means in some studies and medians in others. We did not specify this information for better readability. N/A = not available (no access to the information), NS = not specified (information was not found in the publication)

## Discussion

The aim of this study was to provide a detailed evaluation of the clinical profiles, perioperative outcomes, and short-term survival following different extents of surgical intervention—biopsy, subtotal resection, and GTR—in patients with ISCM. Evidence-based surgical strategies remain challenging, and our study contributes critical insights to the ongoing debate regarding optimal management.

### Surgical extent and survival

Our comparative analysis underscores the nuanced balance between aggressive surgical intervention and patient-specific conditions. Notably, patients undergoing GTR exhibited the lowest early mortality (13% at 90 days) compared to subtotal resection (60%) and biopsy-only (33%). This might suggest superior survival with aggressive tumor removal. However, deeper analysis reveals crucial selection biases: GTR-eligible patients had higher KPS scores (mean KPS 75% vs. 54–63% in other groups). Additionally, biopsy-only cases typically had disseminated disease (e.g., leptomeningeal spread or multiple lesions), limiting their prognosis and treatment options. Logistic regression showed that EOR was not independently associated with three-month survival (*p* = 0.834), indicating survival benefits likely reflect favorable baseline profiles rather than surgical extent. This underscores the confounding impact of patient selection bias in ISCM surgical outcomes and aligns with emerging literature emphasizing individualized patient factors.

A recent systematic review examining ISCM from lung carcinoma similarly concluded that GTR did not provide significant survival advantages over subtotal resection; rather, surgical intervention itself—regardless of radicality—was beneficial compared to non-operative approaches [12]. This review highlighted that symptomatic relief and functional preservation from surgical decompression had greater prognostic relevance than the extent of tumor removal. Correspondingly, Gazzeri et al. also reported no significant survival difference between subtotal and total resections for ISCM, emphasizing symptom management and neurological stability as key outcome drivers [11]. Our results further stress the importance of individualized surgical decision-making. Aggressive GTR may offer benefits in selected cases with isolated lesions and good neurological status, as maximal resection can potentially reduce symptom burden and local disease progression. Conversely, in patients with extensive metastases or poor neurological function, aggressive surgery offers limited clinical benefit. In such scenarios, limited decompression or diagnostic biopsy to preserve function and reduce perioperative morbidity appears more prudent.

### Prognostic factors for survival

Our analysis did not demonstrate significant associations between survival and commonly considered prognostic factors such as age, sex, preoperative Karnofsky score, or neurological grade. While these factors are typically prognostic across oncology populations, the absence of measurable impact in our cohort likely reflects advanced cancer stages and the overall poor prognostic of ISCM. The small sample size further limits conclusions and underlines the necessity for cautious interpretation. Nonetheless, literature reveals several prognostic factors consistently associated with survival following ISCM surgery. Notably, surgical intervention itself emerges repeatedly as a critical determinant of improved survival compared to conservative management. Dam-Hieu et al. reported significantly longer median survival in surgically treated patients (7.4 months) vs. non-surgically managed ones (2.6 months) [20]. Similarly, Goyal et al. observed a doubling in median survival - from 3 to 6 months, with surgical intervention, particularly in patients with solitary metastases and no brain metastases [19]. Wu et al. also identified surgery as an independent protective factor for survival in lung cancer-associated ISCM, underscoring the benefit of surgical decompression in specific subgroups [12].

Additionally, postoperative neurological improvement consistently appears as a strong prognostic marker: Wu et al. (2022) found significant survival advantages linked explicitly to postoperative neurological symptom relief (HR = 0.212, *p* < 0.001), indicating that decompression and functional preservation might outweigh EOR as the key outcome drivers [12]. These findings highlight symptom management and neurological function preservation as central surgical goals. Other relevant factors related include disease extent and MRI-based tumor characteristics. A retrospective study of 61 patients treated with radiotherapy found longer survival when only one spinal cord segment was involved [30]. Similarly, extensive T2 hyperintensity (≥ 3 segments) and multiple intramedullary lesions correlated with shorter median survival, illustrating the potential prognostic relevance of MRI features [14]. These imaging biomarkers may aid clinical decision-making and treatment stratification. Tumor histology findings remain inconsistent, likely due to methodological differences and small cohorts. Lung cancer histology was associated with worse prognosis in one analysis [11], whereas melanoma metastases appeared to predict comparatively favorable survival in another series [27]. Such discrepancies highlight the complexities in evaluating histological impacts within limited patient samples.

In our cohort, analysis of primary histology and adjuvant therapy impact was not feasible due to small patient numbers. However, given the frequent presence of brain or leptomeningeal metastases, these additional metastatic burdens undoubtedly influenced survival. While previous studies found meningeal involvement [11] and number of metastatic organs [27] to be non-significan, these findings warrant careful interpretation due to their limited statistical power. In summary, our findings and the broader literature emphasize surgical decompression and symptom relief as primary prognostic factors in ISCM management, surpassing EOR. Larger-scale studies remain critical to identify and validate prognostic markers and guide more precise, evidence-based surgical strategies for this challenging patient population.

### Perioperative complications and neurological deterioration

One of the most important findings of our study was the low perioperative risk: Our cohort experienced neither intraoperative mortality nor severe complications. Postoperative complications were manageable, such as one wound infection, successfully treated with antibiotics and debridement. Furthermore, we observed no permanent neurological deficits linked to surgery. These favorable results reflect substantial advances in neurosurgical techniques, intraoperative neurophysiological monitoring, and preoperative imaging, all of which have markedly improved ISCM resection safety.

Contextualized against existing literature, our findings support the current safety of surgical approaches: Gazzeri et al. [11, 13] reported neurological deterioration in 20% of their 30-patient multicenter cohort post- ISCM surgery, highlighting that neurological risks, though reduced, are not absent. This underscores the need for meticulous planning, patient selection, and intraoperative caution. Historically, Hejazi & Hassler (1998) examined 80 patients undergoing intramedullary tumor resection, noting neurological improvement in most (63/80) patients. However, among 5 metastatic cases, 60% deteriorated, illustrating the heightened risk of neurological impairment with metastatic vs. primary spinal cord tumors [25]. Such data reinforce the considerable advancements made in neurosurgery, as modern studies now report lower complication rates and improved outcomes.

Nonetheless, complications—particularly CSF leaks and infections—remain relevant and require postoperative vigilance. Several authors reported postoperative complications such as CSF leaks and wound infections [21, 26]. Conversely, most recent studies show minimal surgical complications. Multiple series report no surgical complications, emphasizing advances in (peri-)operative care [12, 22, 23, 28, 29]. Collectively, our data and the literature highlight reduced morbidity and enhanced surgical safety. Nevertheless, surgical decisions must carefully balance benefit with residual risks of neurological decline and other complications. Continued advancements and broader experience will further refine patient selection and techniques, enhancing ISCM surgical safety.

Recent series demonstrate that carefully selected patients can achieve favorable neurological and functional outcomes [27, 28]. Modern studies report that the majority of patients experience neurological preservation or improvement postoperatively: for example, one multicenter analysis noted symptom improvement in ~ 60% of cases (with partial recovery of motor/sensory function and pain relief) and deterioration in only ~ 20% [11] and all patients who were ambulatory preoperatively remained ambulatory after surgery in another cohort [27]. New permanent neurologic deficits are relatively infrequent – permanent postoperative worsening is observed in roughly 10–20% of patients across studies [11, 27]. Importantly, outcomes are influenced by patient and tumor factors: those with milder preoperative deficits and lesions in favorable locations (e.g., dorsally located or cervical tumors) tend to have better recovery, whereas severe preoperative paralysis or tumors involving the thoracic cord portend poorer neurologic improvement [31].

### Integrating systemic therapy and surgery in the management of ISCM

In light of modern oncologic therapies, surgical indications for ISCM must be framed within the patient’s overall disease context. Improved systemic treatments – including chemotherapy, targeted agents (EGFR/ALK inhibitors in lung cancer, HER2-directed therapy in breast, BRAF/MEK inhibitors in melanoma), immunotherapies (checkpoint inhibitors), and advanced radiotherapy techniques – have modestly extended survival in patients with spinal cord metastases and thus influence the role of surgery [32, 33]. Our series of 16 ISCM patients underscores that surgery is most beneficial when the patient’s systemic cancer is controllable or in remission, allowing them to actualize the gains of neurological preservation. Consistent with the literature, we found that carefully selected patients who underwent resection experienced neurological improvement and a meaningful survival interval, whereas those with aggressive, refractory disease derived limited benefit [12, 13]. The availability of effective systemic options can tip the risk–benefit balance in favor of surgery – for example, resecting an intramedullary metastasis in a lung cancer patient responding to an EGFR inhibitor can prevent paralysis and yield survival beyond historically grim expectations [12]. Conversely, in patients with disseminated malignancy lacking further treatment lines, a less invasive approach (such as palliative stereotactic radiosurgery or best supportive care) may be prudent. Notably, emerging modalities like spinal stereotactic radiosurgery now offer a non-surgical means to obtain local control in select cases, and retrospective data suggest combining surgery with adjuvant radiation can maximize disease control for those fit enough [32]. Ultimately, integrating systemic therapy with local treatment is critical: surgery should be seen as one component of a multimodal strategy, deployed when it aligns with the patient’s oncologic trajectory. This nuanced approach – selecting surgical candidates based on tumor biology, systemic therapeutic options, and performance status – is essential for optimizing outcomes in ISCM, as our findings illustrate. In practice, a multidisciplinary assessment (neurosurgery, oncology, radiation oncology) is warranted for each case, ensuring that surgical intervention is offered when it meaningfully improves neurological function and complements the patient’s overall cancer management [34–36].

### Limitations and future directions

This study’s primary limitations are its retrospective design and small sample size (16 patients), limiting statistical power to detect subtle but clinically relevant prognostic associations, such as those related to Karnofsky Performance Status (KPS), tumor volume, or preoperative neurological function. The absence of prognostic significance for these factors should be interpreted cautiously, as larger cohorts may reveal associations missed here. Also, key prognostic factors—such as leptomeningeal progression, CSF cytology, systemic disease burden, and local recurrence timing—could not be consistently assessed. Prospective data collection and structured follow-up would allow deeper insight into their impact on surgical candidacy and outcomes. Selection bias is inherent due to the non-randomized nature of our methodology; surgical strategies (biopsy, subtotal, or total resection) were based on clinical judgment and patient-specific factors rather than prospective allocation. This likely confounds direct outcome comparisons, as patients undergoing aggressive surgery differed in baseline status from those receiving conservative treatment. In this context, tumor histology and systematic postoperative adjuvant treatments may confound these survival analyses. Future studies should employ multicenter collaborations and prospective registries to enhance statistical power and better define prognostic factors. Larger-scale studies stratified by tumor type and clearly defined treatment regimens would also be needed to specifically draw conclusions regarding survival outcomes between GTR and STR approaches. Identifying which patients benefit most from aggressive surgery versus conservative or systemic approaches, and incorporating standardized assessments of quality of life and neurocognitive function, remains a critical goal given the significant functional impact of ISCM.

## Conclusions

Surgical management of ISCM is safe and effective in selected patients, offering symptom relief or functional stabilization with minimal risk of irreversible neurological damage. Although GTR correlated with better short-term survival in unadjusted analyses, this likely reflects selection bias rather than a true survival benefit. Our findings underscore the importance of postoperative neurological status, symptom improvement, and systemic disease burden as key prognostic factors. Surgical decisions should be individualized, prioritizing neurological preservation and quality of life, particularly in patients with solitary lesions and controlled systemic disease.

## Data Availability

No datasets were generated or analysed during the current study.

## Abbreviations

ALL: Acute lymphoblastic leukemia
ASA: American Society of Anesthesiologists
BMI: Body Mass Index
CCI: Charlson Comorbidity Index
EOR: Extent Of Resection
GTR: Gross Total Resection
HR: Hazard Ratio
ICU: Intensive Care Unit
ISCM: Intramedullary Spinal Cord Metastasis
KPS: Karnofsky Performance Score
mMCS: Modified McCormick Score
MRI: Magnetic Resonance Imaging
MS: Motor Score
SD: Standard deviation
